# Evaluating deep learning techniques for optimal neurons counting and characterization in complex neuronal cultures

**DOI:** 10.1007/s11517-024-03202-z

**Published:** 2024-10-17

**Authors:** Angel Rio-Alvarez, Pablo García Marcos, Paula Puerta González, Esther Serrano-Pertierra, Antonello Novelli, M. Teresa Fernández-Sánchez, Víctor M. González

**Affiliations:** 1 Computer Science Department, University of Oviedo, Oviedo, Spain; 2 Biochemistry and Molecular Biology Department, University of Oviedo, Oviedo, Spain; 3 Psychology Department, University of Oviedo, Oviedo, Spain; 4 Electrical Engineering Department, University of Oviedo, Oviedo, Spain; 5 University Institute of Biotechnology of Asturias (IUBA), University of Oviedo, Oviedo, Spain; 6 Biomedical Engineering Center (BME), University of Oviedo, Oviedo, Spain

**Keywords:** Neuron characterization, Instance segmentation, Semantic segmentation, Object detection

## Abstract

**Abstract:**

The counting and characterization of neurons in primary cultures have long been areas of significant scientific interest due to their multifaceted applications, ranging from neuronal viability assessment to the study of neuronal development. Traditional methods, often relying on fluorescence or colorimetric staining and manual segmentation, are time consuming, labor intensive, and prone to error, raising the need for the development of automated and reliable methods. This paper delves into the evaluation of three pivotal deep learning techniques: semantic segmentation, which allows for pixel-level classification and is solely suited for characterization; object detection, which focuses on counting and locating neurons; and instance segmentation, which amalgamates the features of the other two but employing more intricate structures. The goal of this research is to discern what technique or combination of those techniques yields the optimal results for automatic counting and characterization of neurons in images of neuronal cultures. Following rigorous experimentation, we conclude that instance segmentation stands out, providing superior outcomes for both challenges.

**Graphical abstract:**

Identifying the optimal pathway for characterizing neurons in complex cultures through structured experimentation
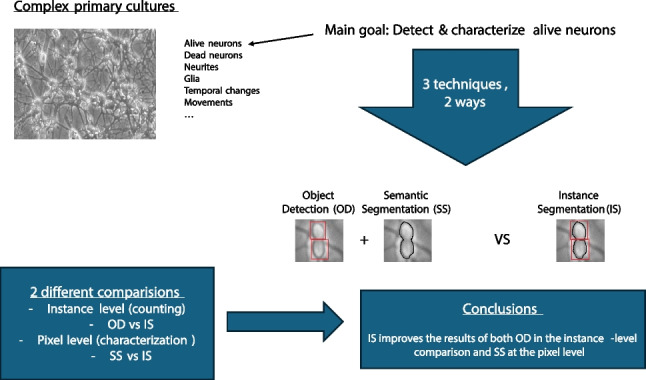

## Introduction

Neurons are the cells primarily responsible for the ability to process and learn information. These cells have a body, known as the soma, and are designed to receive information from other neurons through their dendrites. Neurons can generate and transmit electrical signals, enabling them to induce motion in, for instance, the human body. Within the brain, neurons establish chemical communications among themselves, with their location determining their specific function. In humans, the connections and interactions of neurons play a pivotal role in shaping personality. However, alterations in neurons can manifest as various behavioral symptoms if they malfunction or are influenced by external chemical substances [1].

**Fig. 1:**
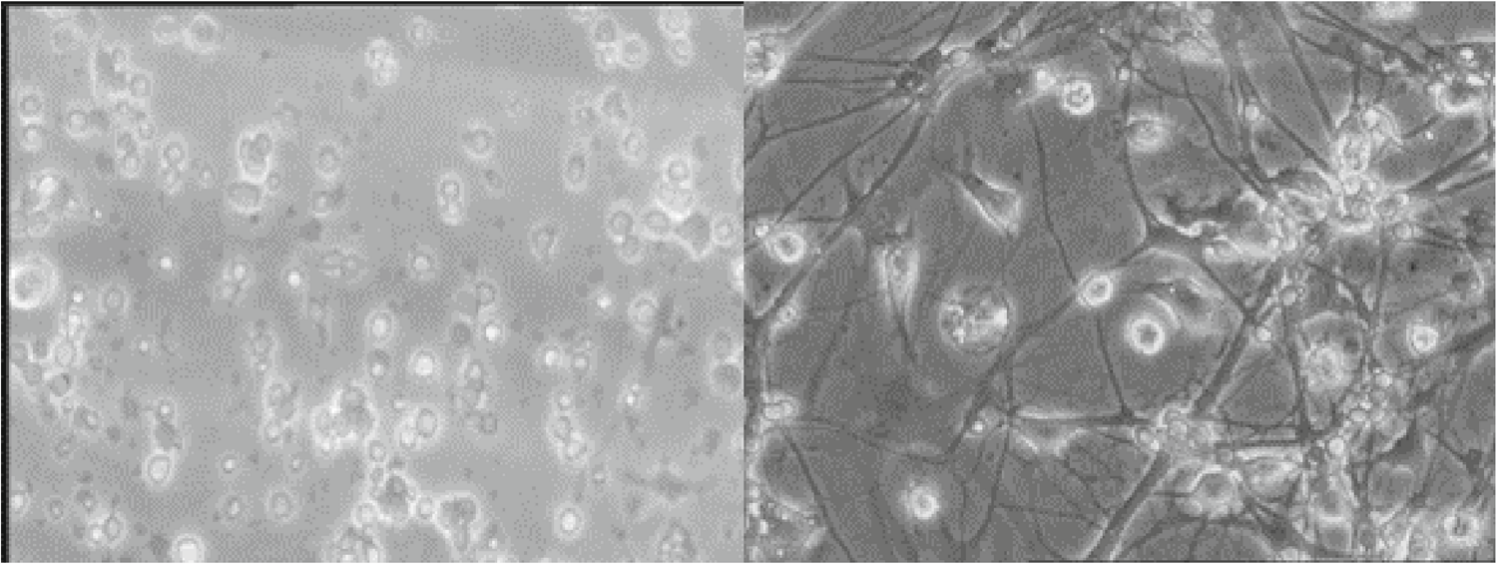
Phase contrast microscopy images of neuron cultures at different times. The image on the left corresponds to an early stage of the cell culture. Neurons establish multiple connections between them over time (right)

Neuronal cell cultures are excellent model systems in a variety of areas of neuroscience research: monitoring of neuronal development, tracking brain changes over time and in response to different stimuli, mechanisms that participate in brain damage and recovery, biosafety and neurotoxicity testing studies, etc. An accurate estimation of cell numbers and the characterization of neurons (assessment of size, shape, and other morphological traits) are essential for these fields of research. Traditional neuron counting techniques, nonetheless, often employ dyes, which can harm or modify the neurons. Therefore, counting and characterizing neurons in a culture at multiple points in time is particularly challenging [2]. Consequently, research studies that aim to monitor the culture’s progression over time are often reliant on manual neuron counting, a process that is not only time-consuming and tedious but also demands specialized expertise. In addition, this method presents its own set of challenges. Neuron cultures contain elements other than neurons, such as glial cells, neurites, dead neurons, and fragments of these structures. Besides, neuron cultures can undergo significant changes over time as neurons move and establish connections among them (Fig. 1). On top of that, dead neurons often cluster with other cells (both living and deceased), complicating their differentiation from living ones [3]. As a result, this complexity makes the identification of living neurons challenging. Experts can distinguish living neurons from other components in the culture based on morphological traits, such as shape, light reflection, and membrane permeability. However, variations in counting criteria can introduce inconsistencies in the counting process. In order to mitigate methodological errors, it is preferable to have multiple experts conducting the counting, but at the cost of more time to obtain results.

The development of new techniques for counting and characterizing the number of neurons in neuronal cultures could profoundly benefit biomedical research. Identifying a technique capable of accurately counting and characterizing neurons without damaging the culture or overburdening experts would enhance our research capabilities in areas like brain behavior, neurodegenerative diseases, and the effects of potentially harmful substances on the brain. In this sense, traditional techniques, which primarily rely on staining methods and manual segmentation [4, 5], have been complemented in recent years by non-invasive computer vision-based approaches. Specifically, techniques rooted in deep learning (DL) have heralded a transformative shift in this domain. DL has seen substantial growth, driven by enhanced computing capabilities and the availability of vast datasets for training models [6]. One prominent area within DL is computer vision, an artificial intelligence domain that enables computers to extract meaningful information from digital images, videos, or other visual inputs [7]. Recent advancements in computer vision have demonstrated state-of-the-art results [8]. Given the prowess of computer vision models and the need for efficient neuron counting and characterization, it seems plausible to develop a model that can process images of neuronal cultures and produce relevant information about it, such as the number of neurons, dead and alive, and their characteristics.

After an intensive analysis of the state of the art, we determined that the two main strategies offered by computer vision to develop complex algorithms for extracting the desired information from the neuronal culture images are (i) object detection (OD) (to count and to locate neuron’s positions) combined with semantic segmentation (SS) (to identify neuron’s shape) and (ii) instance segmentation (IS) (to count neurons, to locate position, and to identify shape). Although IS provides the tools needed to address all the challenges of this problem, it is not obvious that it always outperforms the other strategy as it is a highly context-dependant technique [9].

These two strategies are the foundation of any comprehensive deep learning-based method for extracting information from images. Therefore, determining which one is the most suitable to process this type of image is key to deciding what strategy to adopt to address the problem stated in this research, and for future more complex methods to develop.

The subsequent sections of this paper are structured as follows: Sect. 2 offers an overview of state-of-the-art computer vision techniques; Sect. 3 details the architecture of the models used as well as their formal connection to our hypotheses; Sect. 4 details the experimental procedures employed to assess the viability of the discussed techniques; Sect. 5 presents and deliberates on the experimental results; Sect. 6 concludes the paper, suggesting potential avenues for future research; and finally, Sect. 7 explains the application limits of our work and conclusions.

**Fig. 2:**
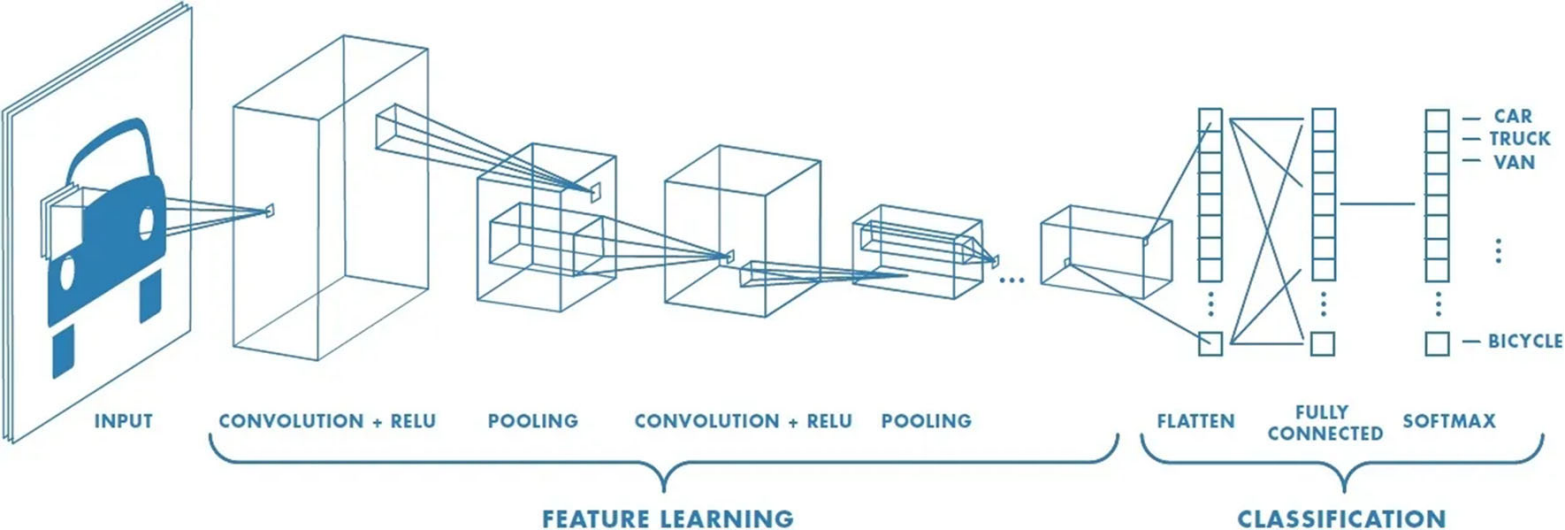
Convolutional neural network diagram [29]

## Background

Image-based cell analysis is essential in biomedical research, particularly in studying cell morphology, cell behavior in culture, and cell tracking [10–12]. Manual analysis of large quantities of images is not sustainable. Thus, automated image processing techniques are needed. These new techniques require reliable tools for automatic quantitative analysis of neuronal cultures. Existing automatized cell recognition methods often have limitations, such as using only a few features and being designed only for fluorescence microscopy images [13].

Microscopy image analysis tools employ two main methods: image segmentation and image classification. Segmentation methods group pixels based on their similarities, distinguishing image objects from the background [14, 15]. Classification methods assign labels to images or specific areas within images for object recognition [16, 17]. Both approaches have proven useful in identifying normal and abnormal cells in microscopic images [18]. Image classification is typically implemented in microscopy images through object detection (OD), which is a technique that combines classification with a sliding window, allowing to identify objects in different parts of the image [19]. Image segmentation, on the other hand, can be implemented as semantic segmentation (SS) if we only want to assign a label to each pixel in the image, or as instance segmentation (IS), which, in addition to labeling each pixel, can recognize the different instances of the same class as in the case of object detection [20]. Due to the requirements of this study, such as cell morphology recognition and localization, these three techniques, SS, OD, and IS will be studied in this research.

### Semantic segmentation

Semantic segmentation involves classifying each pixel in an image into a predefined set of classes [21]. This technique is primarily useful for characterizing neurons based on their morphological traits but does not directly provide a neuron count. Traditional models for this task relied on hand-crafted features, but they often faltered in the presence of object occlusions, a common occurrence in neuronal cultures. Recent advances have seen convolutional neural networks (CNNs) enabling semantic segmentation models to automatically extract features from images with groundbreaking improvements [21]. CNNs are a type of artificial neural network designed to process data that comes in the form of multiple arrays (Fig. 2). These networks operate through convolutional layers (for feature extraction), non-linearity layers (introducing non-linearities), pooling layers (for down-sampling), and fully connected layers (for final predictions) [22].

Deep learning approaches, particularly CNNs, have shown remarkable success in semantic segmentation tasks across various domains [23]. Architectures such as U-Net have been used for biomedical image segmentation in various works such as the approach made by Ibtehaz and Sohel Rahman [24]. Besides biomedical image, further improvements have been made in recent years, contributing to the success of this type of neural network. Chen et al. [25] proposed DeepLab, in the pursuit of creating a more accurate architecture for segmentation. A year later, DeepLab was refined, achieving an improved performance [26]. Aiming to improve speed, Paszke et al. [27] created ENet, a lightweight and efficient architecture aimed at mobile applications. Also improving performance, Chaurasia and Culurciello [28] introduced in LinkNet the concept of Link Blocks to improve the connectivity between the encoder and the decoder of the network.

### Object detection

Unlike semantic segmentation, classification-based approaches can detect cell overlapping and distinguish different cell shapes and types [30]. OD focuses on classifying and pinpointing objects in images or videos [31]. This method has been the subject of increased interest in research. In 2014, SPP-Net was presented using a Gaussian pyramid pooling layer after the last convolutional layer, allowing the net to handle images of different sizes [32]. The same year, GoogleNet offered a new architecture, performing parallel convolutions with different scales of the images [33]. In 2016, single shot multibox detector (SSD) was introduced, a fast and accurate OD model that uses pre-defined bounding boxes as reference points for predicting locations and shapes [34]. 2017 saw the birth of Densenet, which attempts to improve performance by connecting all layers between them [35]. Also in 2017, a feature pyramid network was introduced, being the first model to create a feature pyramid by fusing features from different levels [36]. This year also saw the presentation of RetinaNet, introducing a reshaping standard cross-entropy function to downweight the impact of certain classes, aiming to improve their classification performance [37]. In 2018, CornerNet was proposed as a one-stage detection model that recognizes the region of interest from the input samples through key point calculation, offering accurate detection [38]. object detection is widely used for counting neurons. Various methods, both traditional and based on deep learning have been proposed for this task [39]. In 2015, the first version of the YOLO architecture was introduced [40]. YOLO represented a revolution in object detection methods because, for the first time, the entire image was processed in a single neural network, unlike previous methods that divided the image into regions. This resulted in a significant improvement in processing speed [41]. Since its creation, YOLO has spawned multiple computational vision models, such as the YOLO v5 and YOLO v7. These models have shown progress compared to the original. For example, YOLO v5’s architecture requires a reduced training time, which, combined with its easier installation, makes it a widely used YOLO variant [42]. An in-depth comparison between YOLO variants was made by Sirisha et al. [43], which provided a detailed overview of the YOLO architecture before analyzing the strengths and weaknesses of each variant.

### Instance segmentation

Instance segmentation has been gaining a lot of interest in recent years [44], as it allows for both counting and characterizing neurons in the culture. This technique, a fusion of semantic segmentation and object detection, assigns unique labels to object instances, enabling distinct treatment of objects even within the same class [45]. This combination involves a more sophisticated and detailed approach. Object detection identifies areas of interest in an image while semantic segmentation assigns labels to each pixel based on predefined categories. In contrast, instance segmentation goes further by providing a unique identification to each individual object, even when they are overlapping. This is achieved through advanced techniques, such as the use of segmentation masks to accurately delineate the contours of each instance. Thus, IS not only allows understanding of what objects are present in an image and where they are located but also provides a detailed understanding of the distribution and interaction of these objects within the visual scene [46].

IS has also been targeted by multiple modern investigations. Tian et al. [47] proposed conditional convolutions, where the convolutional operations are conditioned on the instance characteristics. Meanwhile, Chen et al. [48] presented a different approach by combining instance-level information with semantic information with lower-level fine-granularity. Kirillov et al. [49] introduced an alternative method based on point-based segmentation predictions at locations selected by an iterative subdivision algorithm. Furthermore, Wang et al. [50] proposed a method for instance segmentation that associates object instances with individual pixels, decomposing segmentation into two classification tasks. Another approach was made by Bolya et al. [51], which breaks instance segmentation into two parallel subtasks (one generates a set of prototype masks, while the other makes predictions) achieving speed improvement. In recent years, the YOLO architecture, highly used in object detection, has moved into instance segmentation with versions like YOLO v7, which managed to integrate segmentation efficiently in real-time without significantly sacrificing accuracy or speed [52]. This was achieved through the extension known as YOLOv7-mask, which combines the object detection capabilities of YOLOv7 with precise instance segmentation using advanced re-parameterization techniques and fine-tuning of the model. In this way, the YOLO architecture also becomes one of the most widely used for instance segmentation [53].

**Table 1:** Main characteristics of the techniques considered in this work: semantic segmentation, object detection, and instance segmentation

	Recognition element	Identification process	Ref.	Application in neuronal cultures
Semantic segmentation	Shape at pixel level	Assignation of labels to each pixel based on predefined categories	[23–27]	Characterization of neurons based on morphological traits
Object detection	Number and position of elements	Identification of areas of interest in the image	[31–39]	Counting neurons
Instance segmentation	Number, position, and shape	Unique identification to each individual object	[46–50]	Counting and characterization of neurons

### Background conclusions

Previous comparison studies focused on deep learning models. In the context of semantic segmentation, different variants of U-Net architecture were analyzed [24]. Comparisons between ENet and SegNet were also addressed by Paszke et al. [27] and Chaurasia et al. [28]. Similarly, the literature on object detection and instance segmentation predominantly centers around comparative analyses of similar architectures. As noted, several studies, including Zhang et al. [35] and Lin et al. [36], compared different variants of YOLO or other object detection models against each other. Instance segmentation research, exemplified by Chen et al. [48], often compares against prevalent models like Mask R-CNN (Table 1).

Our work provides a novel approach, by comparing the performance of semantic segmentation with that of instance segmentation, and by comparing object detection and instance segmentation techniques directly against each other. With these objectives in mind, the aim of our study is to resolve the uncertainty surrounding the performance disparity between SS, OD, and IS when confronted with the intricate task of characterizing and enumerating neurons within complex neuronal cultures.

## Characterization of neuronal cultures using deep learning-based strategies

The analysis of primary neuronal cultures, specifically from the rat cerebellum under the described conditions, presents a unique set of challenges due to the complexity and variability of the environment. These cultures include a variety of elements such as astrocytes, cell debris, dead neurons, neurites, and are also highly dynamic and changing over time. Characterizing these neuronal cultures involves not only counting and locating each neuron but also identifying their precise morphology, which is crucial for advanced neuroscientific studies.

To address this problem, deep learning techniques offer two main strategies: the combination of object detection with semantic segmentation [54, 55], and instance segmentation [47–49]. Each approach has its own strengths and limitations, and its effectiveness can vary depending on the specific context of the problem [9]. In this section, we will explore both approaches, analyzing their mathematical bases and determining in which situations each might be more suitable.

### Architectures overview

Before discussing these techniques, we will briefly review the different deep learning neural network architectures used in this research.

YOLO has been chosen as the most representative architecture for object detection and instance segmentation because it has been the most widely used in recent years [56]. YOLO prevents the need for region proposal extraction, directly estimating bounding boxes and class probabilities from whole images instead. It segments images into grids, predicts object locations, and applies non-maximal suppression to eliminate redundant detections [57, 58]. In order to select the most representative architecture for the semantic segmentation model, U-Net has been chosen because it is the most widely used architecture for microscopy images [59].

**Fig. 3:**
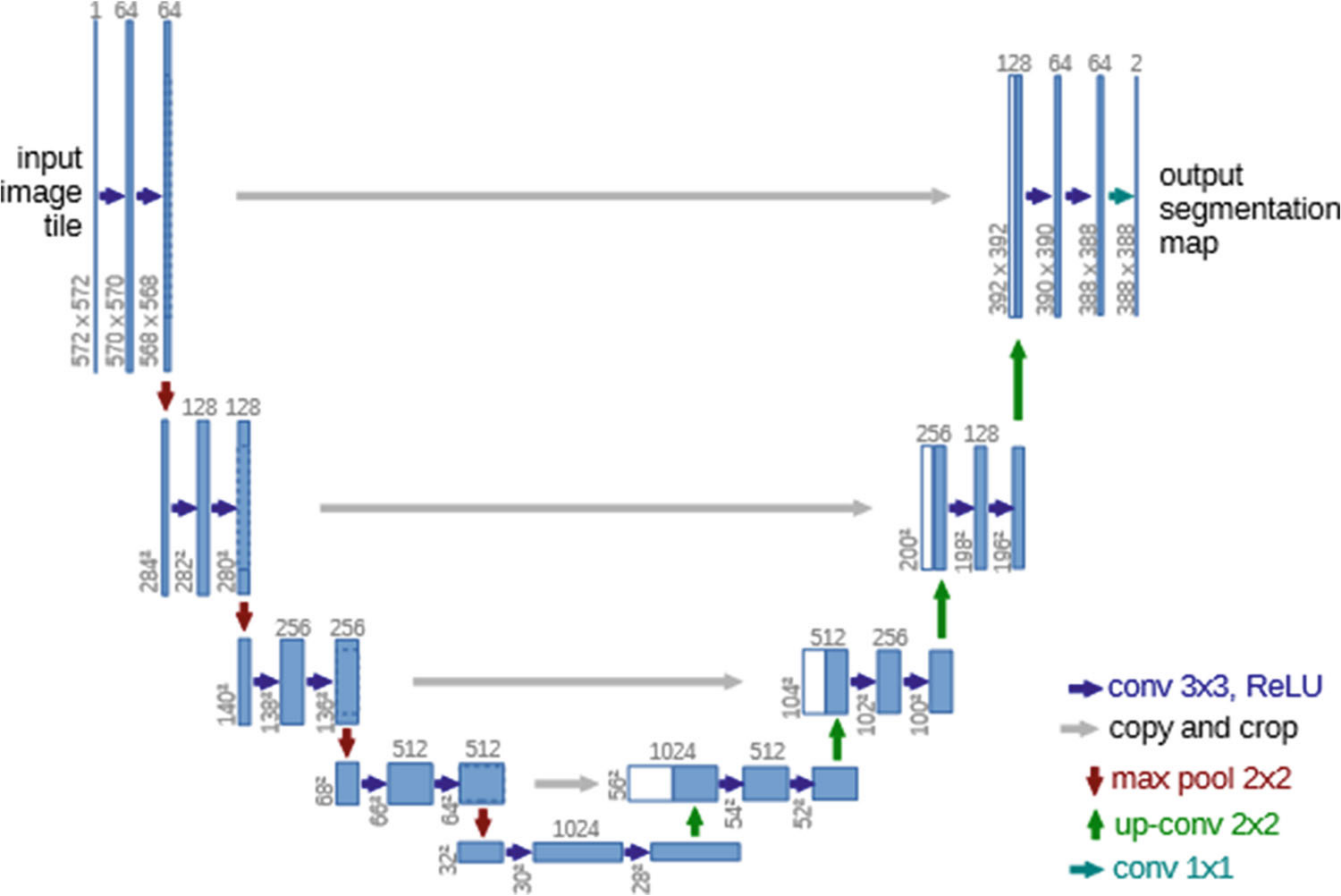
U-Net architecture displaying the layers used to process the input [60]

U-Net, introduced in 2015, enhances semantic segmentation, particularly in biomedical imaging. This network, named for its U shape, comprises two main components: a contracting path (encoder) and an expansive path (decoder), as seen in Fig. 3. The encoder path reduces the spatial dimensions of the input images while capturing features through convolutional and pooling layers to down-sample the data. The decoder path aims to restore the original resolution of the input images, using up-sampling techniques and transposed convolutions to produce a high-resolution segmentation map [60].

During the up-sampling process in the decoder path, feature maps from corresponding layers of the encoder path are concatenated with the up-sampled output. This process incorporates localized information, facilitating more precise and accurate segmentation.

**Fig. 4:**
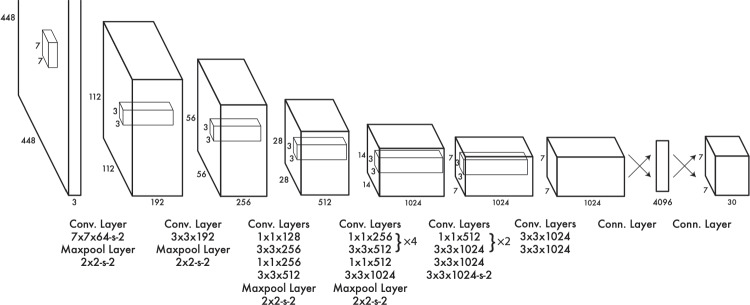
YOLO architecture showing the layers used to process the input [40]

YOLO, introduced in 2016, detects objects as a single regression problem, directly predicting bounding boxes and class probabilities from the entire image in one evaluation [40].

The YOLO architecture, as seen in Fig. 4, divides the input image into a grid of cells. Each cell predicts a fixed number of bounding boxes and their corresponding confidence scores. These confidence scores reflect the likelihood that a bounding box contains an object and the accuracy of the bounding box prediction. YOLO also predicts the class probabilities for each detected object, enabling the simultaneous identification and localization of multiple objects within an image.

### Object detection combined with semantic segmentation strategy

Object detection, in this research implemented by YOLO [40], is a technique that divides the image into a grid and for each cell predicts bounding boxes and class probabilities. YOLO uses a single convolutional network that processes the entire image to predict bounding boxes and object classes simultaneously. The loss function for YOLO (1) is a combination of the bounding box regression loss $L_{box}$, the classification loss $L_{cls}$, and the confidence loss $L_{conf}$:

1 $$\begin{aligned} L = L_{box} + L_{cls} + L_{conf} \end{aligned}$$

where $L_{box}$ ensures that the bounding boxes fit the detected neurons properly, $L_{cls}$ ensures that the neurons are classified correctly, and $L_{conf}$ improves the confidence in the detections made. OD is particularly effective in contexts where objects are relatively isolated [61]. The combination of $L_{box}$ and $L_{conf}$ provides a rapid identification and location of neurons using bounding boxes, which is sufficient for counting and locating tasks in less dense regions.

Semantic segmentation using networks like U-Net [60] assigns a class label to each pixel in the image, differentiating the areas occupied by neurons from other elements. U-Net has an encoder-decoder structure that allows for precise segmentation using convolution and deconvolution operations. The loss function in SS is typically cross-entropy (2), defined as:

2 $$\begin{aligned} L_{seg} = -\sum _{i} \left[ y_i \log (\hat{y}_i) + (1-y_i) \log (1-\hat{y}_i) \right] \end{aligned}$$

where $y_i$ is the true label and $\hat{y}_i$ is the predicted probability for pixel $i$.

This loss function $L_{seg}$ ensures that each pixel is correctly labeled, allowing for detailed segmentation of the areas of interest. SS in combination with OD provide precise segmentation of the regions of interest, enabling the analysis of the general morphology of neurons. However, this combination is less effective in areas with high cell density and overlaps, where detailed segmentation precision is crucial, as the $L_{seg}$ loss function may not adequately capture the differences between overlapping neurons.

### Instance segmentation strategy

Instance segmentation is not just object detection combined with semantic segmentation; rather, both processes are interconnected, and the information from each one is utilized by the other to enhance their efficiency [55]. In this research, YOLOv7 has been used. It is a specific version of YOLO for IS that extends the YOLO architecture to not only detect objects but also to segment each individual instance. YOLOv7 optimizes a combined loss function (3) that includes terms for classification $L_{cls}$, bounding box regression $L_{box}$, and segmentation $L_{mask}$:

3 $$\begin{aligned} L = L_{cls} + L_{box} + L_{mask} \end{aligned}$$

In this formula, $L_{cls}$ and $L_{box}$ function similarly to how they do in object detection, ensuring correct identification and location of neurons. The addition of $L_{mask}$ allows for precise pixel-level segmentation for each detected neuron:

4 $$\begin{aligned} L_{mask} = -\sum _{i,j} \left[ M_{ij} \log (\hat{M}_{ij}) + (1-M_{ij}) \log (1-\hat{M}_{ij}) \right] \end{aligned}$$

where $M_{ij}$ is the true mask value for pixel $(i,j)$ and $\hat{M}_{ij}$ is the predicted value.

The loss function $L_{mask}$ ensures that each pixel within a bounding box is correctly segmented, allowing differentiation between individual neurons even when they are overlapping. This is crucial in areas with high density and complexity, where neurons can have irregular shapes and be overlapped. IS, by jointly optimizing classification, bounding box regression, and pixel segmentation, allows detailed segmentation of each neuron, facilitating the analysis of their precise morphology and exact location.

Due to the multiple characteristics that make one approach better than the other depending on the specific context, it is not obvious the selection of one of both possibilities [9]. In certain contexts, the combination of object detection and semantic segmentation can be beneficial, while in others, instance segmentation may offer better results. The importance of this work lies in shedding light on which strategy to follow in the specific context of primary neuronal cultures described in the introduction section of this paper. The findings will provide clear guidance for researchers, helping to develop effective and comprehensive solutions for neuron characterization in this kind of complex and dynamic environments. This study could be a crucial starting point for future work in similar cultures, improving the accuracy and efficiency of analysis techniques.

## Materials and methods

In this study, we present a dual comparative approach. Firstly, we compare the capabilities of semantic segmentation and instance segmentation in providing detailed morphological data about neurons. Secondly, we contrast object detection and instance segmentation in their proficiency for neuron recognition. While both comparisons offer valuable insights, our overarching objective remains to evaluate the relative efficacy of these techniques in recognizing, counting, and characterizing neurons within our intricate cultures.

To conduct this research, we made our own dataset of neuronal cultures annotated by experts. We also had to choose the models to be generated as well as the metrics with which to carry out an exhaustive comparison.

**Fig. 5:**
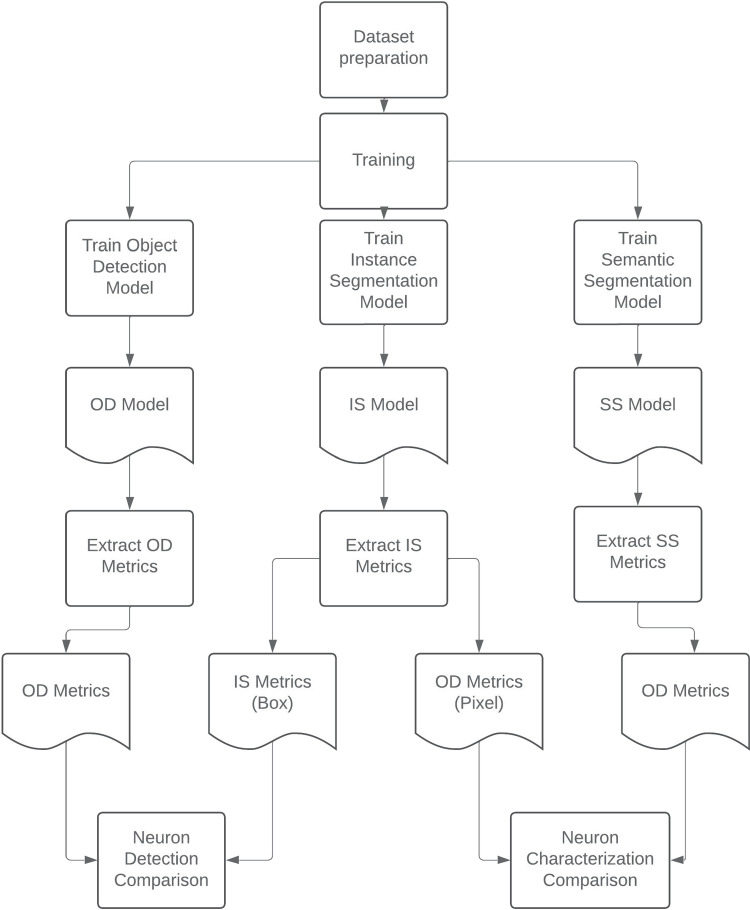
Experimentation process diagram

For a rigorous comparison, a unique and exhaustive experimental protocol was employed. The dataset remained consistent across all generated models, ensuring uniformity in experimental conditions. Additionally, identical experimentation protocols were applied in both comparisons, alongside the utilization of identical evaluation methods and metrics. As shown in Fig. 5, the three models necessary to carry out both comparisons were trained with the same dataset. All the required metrics were obtained by running these models under exactly the same conditions on a common test dataset. Once all the metrics were computed, the two comparisons, which are the focus of this research, were performed.

All the processes, models, and metrics presented in Fig. 5 will be explained throughout this section.

### Dataset

The dataset is made up of a collection of 292 images of primary rat cerebellar neuronal cultures. The dataset was divided as follows: 205 images (70%) for training, 58 images (20%) for validation, and 29 images (10%) for testing. These images, representing different stages of cultures’ development, were obtained using a standard phase-contrast microscope, a Olympus IMT-2 model, equipped with a Sony SPT-M308CE video camera.

**Fig. 6:**
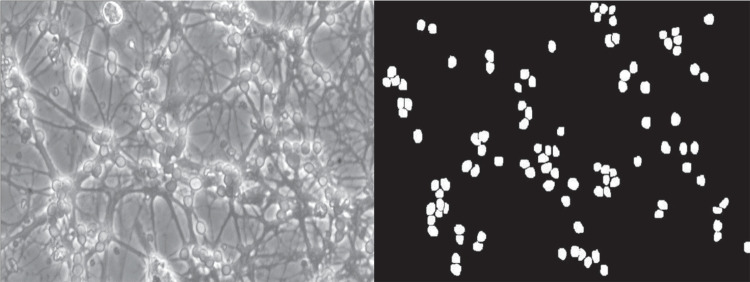
Comparison between an original image (left) and its corresponding mask (right)

Expert biochemists, specialized in neuronal cultures analysis, meticulously annotated each image, ensuring the dataset’s integrity. The annotations were presented as masks, with neurons distinctly marked in white against a contrasting black backdrop. For the purpose of training the object detection model, these masks were converted into bounding boxes. Each image contained 50 neurons on average. A visual representation of the relationship between an image and its corresponding mask is illustrated in Fig. 6.

### Pre-processing

Given the intricacies of our dataset, image preprocessing was imperative to ensure that the input image quality did not compromise model performance. The original images underwent contrast-enhancing filters to accentuate neuronal boundaries. A Gaussian filter was first applied to minimize image noise, followed by a Sobel filter to emphasize neuron edges. For the masks, addressing neuron overlap was crucial, as neurons often clustered without clear demarcations. Untreated masks would inadequately represent cell contours, hindering models from learning accurate cell boundaries in overlapping scenarios. To rectify this, a Voronoi-Otsu-based algorithm [62, 63] was employed, drawing separations between neurons upon detecting tonal shifts induced by the Voronoi-Otsu algorithm itself.

### Deep learning models

In order to take advantage of the capabilities of deep learning for neuron counting and characterization within complex neuronal culture images, we have employed several leading-edge techniques. Initially, for the task of object detection, we utilized the YOLO v5 model, which has consistently showcased superior performance across a range of applications [64].

For the task of semantic segmentation, we turned to the U-Net model. This model provides pixel-level metrics that offer insights into the overall distribution and presence of neuronal structures [65].

The decision to employ YOLO v5 for object detection and U-Net for semantic segmentation is grounded in numerous studies [66–69] that advocate for these technologies as the most representative of each technique when it comes to comparisons or complementary applications. For instance, a study on road crack detection demonstrated the effectiveness of the YOLO v5 model for object detection and the U-Net structure for segmentation, achieving an impressive accuracy of 99.35% [70]. Such findings underscore the prominence and reliability of these models in diverse applications, further justifying their selection for our research.

Lastly, for the comprehensive task of instance segmentation we employed the YOLO v7 model. This decision was influenced by recent studies, such as the one on the instance segmentation of standing tree images, which highlighted the novelty and effectiveness of YOLO v7 in this domain. The YOLO v7 algorithm boasts superior detection speed and accuracy compared to its predecessors [71]. Furthermore, its adaptability, coupled with the addition of the attention mechanism, makes it exceptionally suitable for intricate tasks like the semantic segmentation of trees. Given these advancements and the promising results showcased in the literature, YOLO v7 emerged as our preferred choice for implementing instance segmentation in our study.

YOLO v7 is particularly noteworthy as it offers two distinct sets of metrics. The first set, at the pixel level, aligns with the metrics from semantic segmentation, providing insights into the overall neuronal distribution. The second set, at the object level, focuses on individual neuron recognition and characterization, serving as a foundation for comparison with the metrics from object detection [72].

All three required models were trained using the dataset described above, maintaining the same distribution of samples across training, testing, and validation sets. Given the high density of neurons present in each sample, we determine the dataset is sufficiently comprehensive for our purposes.

Through rigorous testing to ascertain the optimal training duration for each model, a U-Net semantic segmentation model was developed after 200 epochs, an instance segmentation model based on YOLO v7 was refined over 65 epochs, and a YOLO v5 object detection model was honed through 200 epochs. These three optimized models were subsequently validated and compared, with the results presented in the subsequent section.

### Metrics

In the realm of our comparative analysis, it is imperative to elucidate certain nuances associated with the metrics employed. While both comparisons might appear analogous on the surface, there exist intrinsic disparities in the metrics used. Metrics grounded on the count of true positives (TP), false positives (FP), true negatives (TN), and false negatives (FN), despite being computed using analogous methodologies, yield results that are inherently non-comparable. Therefore, when we talk about Precision (5), recall (6), accuracy (7) or specificity (8) in the segmentation-level comparison, we are referring to metrics calculated at pixel level, while when we talk about these same metrics in the object-level comparison, we are referring to metrics calculated at object level. The same applies to F1 metric (9), which is calculated directly from precision (5) and recall (6).

**Table 2:** Performance of the models in neuron segmentation

Model	Accuracy	Precision	Recall	Specificity	F1	IoU
Semantic segmentation	0.9710	0.7827	0.8273	0.8348	0.8038	0.8026
Instance segmentation	0.9697	0.9098	0.8895	0.9410	0.8995	0.9152

In both cases, precision (5) quantifies the ratio of true positive detections to all detected positives, whereas recall (6) measures the ratio of true positive detections to all actual positives present in the image. Accuracy (7), on the other hand, measures the proportion of correctly classifications (both positive and negative) out of the total population, providing an overall sense of the model’s performance. Specificity (8), or the true negative rate, quantifies the proportion of actual negatives that are correctly identified, making it crucial for datasets where the distinction between false positives and true negatives is significant. The F1 score (9), as the harmonic mean of precision and recall, balances the trade-off between these two metrics, providing a single metric that captures both aspects of performance. In all cases, the metrics refer to total number of neurons or pixels depending on the strategy used: object-level or pixel-level.

5 $$\begin{aligned} \text {Precision} = \frac{\text {TP}}{\text {TP + FP}} \end{aligned}$$

6 $$\begin{aligned} \text {Recall} = \frac{\text {TP}}{\text {TP + FN}} \end{aligned}$$

7 $$\begin{aligned} \text {Accuracy} = \frac{\text {TP + TN}}{\text {Total Population}} \end{aligned}$$

8 $$\begin{aligned} \text {Specificity} = \frac{\text {TN}}{\text {TN + FP}} \end{aligned}$$

9 $$\begin{aligned} \text {F1 Score} = 2 \cdot \frac{\text {Precision} \cdot \text {Recall}}{\text {Precision} + \text {Recall}} \end{aligned}$$

For the neuron segmentation comparison (pixel-level), the metrics previously described as common to both types of approaches will be used: precision (5), recall (6), accuracy (7), specificity (8), and F1 (9). In addition, we will employ the intersection over union (IoU) metric. As can be seen in Eq. 10, this metric allows us to assess the overlap between the predicted segmentation (*A*) and the ground truth (*B*), providing a more comprehensive understanding of the model’s accuracy in delineating the exact spatial extent of neuronal structures within the image. The IoU quantifies the degree to which the predicted pixels align with the actual pixels of interest, making it an indispensable metric for evaluating the performance of semantic segmentation models.

10 $$\begin{aligned} IoU = \frac{\text {Area of Overlap}}{\text {Area of Union}} = \frac{|A \cap B|}{|A \cup B|} \end{aligned}$$

To perform the comparison at the level of neuron identification (object-level), it is imperative to evaluate metrics at the bounding box level for instance segmentation model to compare them with the metrics obtained from the object detection model. This stems from the capability of IS to not only pinpoint an object’s presence but also to map out its exact location within the image [73]. For this second comparison, in addition to precision (5), recall (6), accuracy (7), specificity (8) and F1 (9), we will employ the mean average precision (mAP) (12) with an intersection over union (IoU) threshold set at 0.5. This metric offers a consolidated score that captures the balance between precision and recall over varying IoU thresholds, making it invaluable for contrasting different model performances [74]. Regarding mAP, as can be seen in Eq. 12, it is typically computed by averaging the AP (average precision) overall classes (11). In Eq. 11, *p*(*r*) represents the precision at recall *r*. In Eq. 12, *C* is the number of classes and $AP_i$ is the average precision for class *i*.

11 $$\begin{aligned} AP = \int _0^1 p(r) \, dr \end{aligned}$$

12 $$\begin{aligned} mAP = \frac{1}{C} \sum _{i=1}^{C} AP_i \end{aligned}$$

This research has obtained several metrics measuring the performance of the three techniques applied.

First, we can measure how efficient our models are at correctly classifying the pixels of each image as either “part of a neuron” or “non part of neuron.” We have two models capable of doing this type of classification: the semantic segmentation and the instance segmentation models. Table 2 displays the accuracy, precision, recall, specificity, F1, and IoU score of both when performing a segmentation task.

## Results

The results displayed by both models allow us to highlight the capability of computer vision models to be an alternative to traditional techniques. As we can see in Table 2, the instance segmentation model outperforms the semantic segmentation one. Accuracy is higher in the instance segmentation model, but as anticipated, this is not a very relevant metric due to the class imbalance. Accuracy is calculated as the percentage of correct predictions over the total predictions made, so it is not very meaningful in these cases.

If we look at the recall, we can see that the instance segmentation model is able to obtain a higher number of positive predictions. Even more relevant for this type of problem is the IoU, which measures the intersection between both sets and is a metric unaffected by class imbalance. As we can see in Table 2, the IoU experiences a significantly meaningful increase in the instance segmentation model, leaving no doubt about which model has better performance when segmenting neurons. Other relevant metrics for this type of segmentation, due to their good performance with imbalanced classes, are Precision and F1. Both metrics show a significant increase in the instance segmentation model compared to the semantic segmentation one. These results have been determined statistically significant after performing the student’s *T*-test with 95% confidence.

**Fig. 7:**
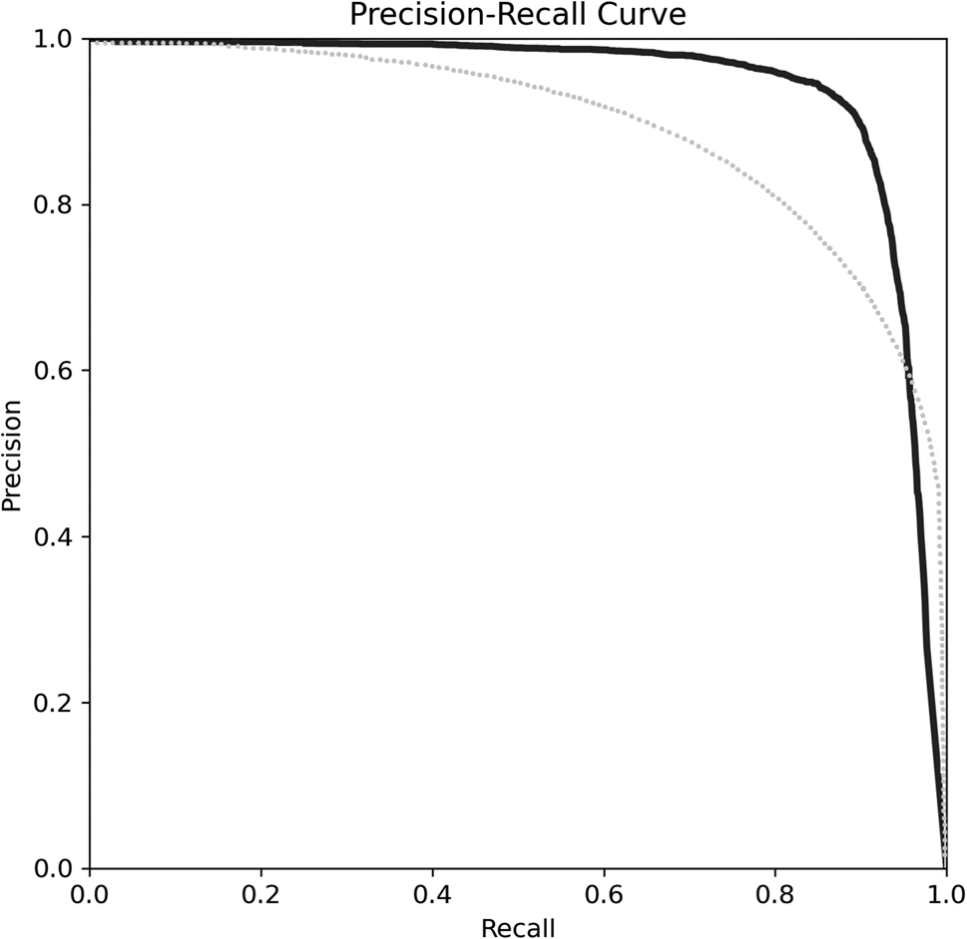
Precision-Recall for Semantic Segmentation (dotted line) and Instance Segmentation (solid line) models corresponding to the Neuron Segmentation comparison

Figure 7 displays the precision-recall curve of both segmentation models. A perfect segmentation would display a precision and recall of 1.0 (the borders of the curve would match perfectly the top and right axis), with the best model being the one closest to it. In the figure, we can see that instance segmentation outperforms semantic segmentation, as its area under the curve is bigger.

**Table 3:** Performance of the models in neuron identification

Model	Accuracy	Precision	Recall	Specificity	F1	mAP@0.5
Object detection	0.9700	0.9217	0.8950	1.0000	0.9081	0.9451
Instance segmentation	0.9750	0.9236	0.9108	1.0000	0.9171	0.9559

Secondly, we can measure how efficient our models are at correctly identifying each neuron in the image. We have two models capable of doing this neuron detection: the object detection and the instance segmentation models. The precision, recall, specificity, F1, and mAP (mean average precision) of these models are shown in Table 3.

Although the differences are small, the trend observed in the previous comparison is repeated in this case. The results obtained through instance segmentation improve on all metrics compared to those obtained through object detection. However, in this case, the precision in both models is similar, metrics such as F1 and the specific metric mAP show significant differences in favor of the instance segmentation model. These differences, once again, were proven to be statistically significant by the student’s *T*-test, demonstrating that at instance-level evaluated, IS model also outperforms the OD model.

Still, both models show results that make them a solid alternative to manually counting neurons or using dyes, as they perform automatically the neuron counting task without damaging the culture or requiring time from domain experts.

**Fig. 8:**
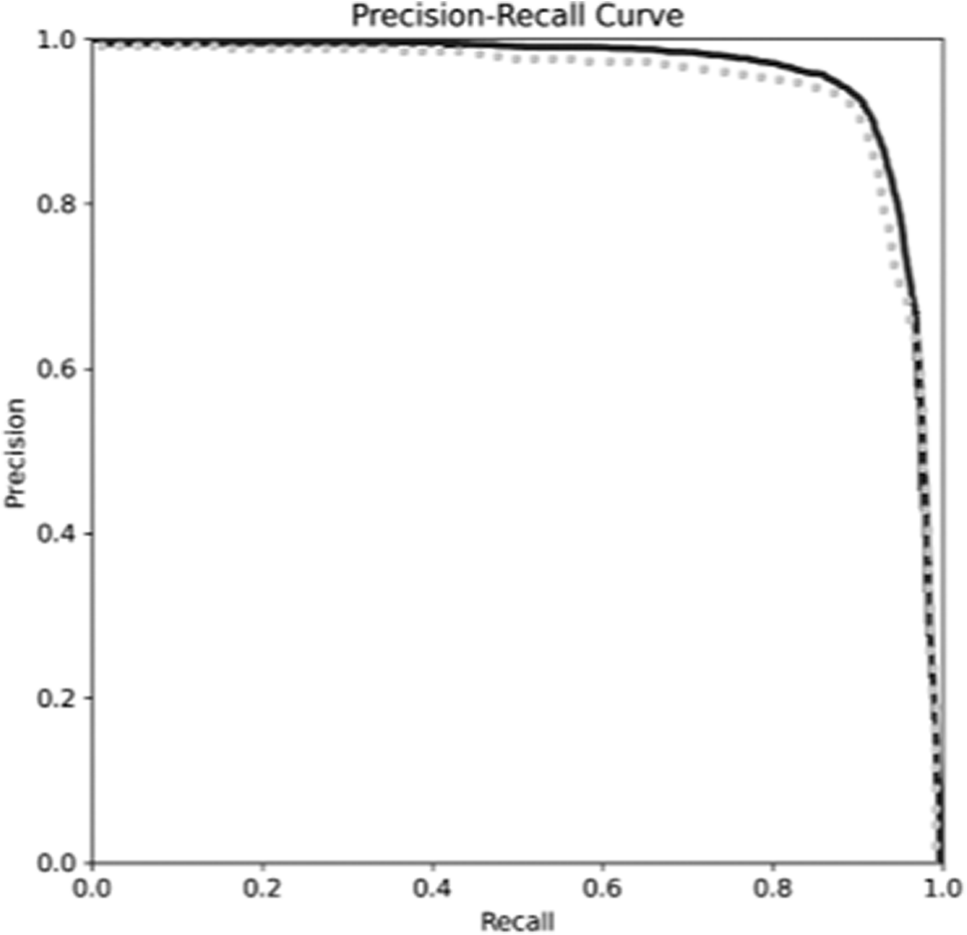
Precision-Recall for Object Detection (dotted line) and Instance Segmentation (solid line) models corresponding to the Neuron Detection comparison

Figure 8 displays the precision-recall curve of both classification models. A perfect classification curve would display a precision and recall of 1.0 (the borders of the curve would match perfectly the top and right axis). It is unlikely that a real model displays a perfect curve, but the best model would be the one closest to it. In the figure, we can see that instance segmentation outperforms object detection, as it is closer to a perfect curve.

**Fig. 9:**
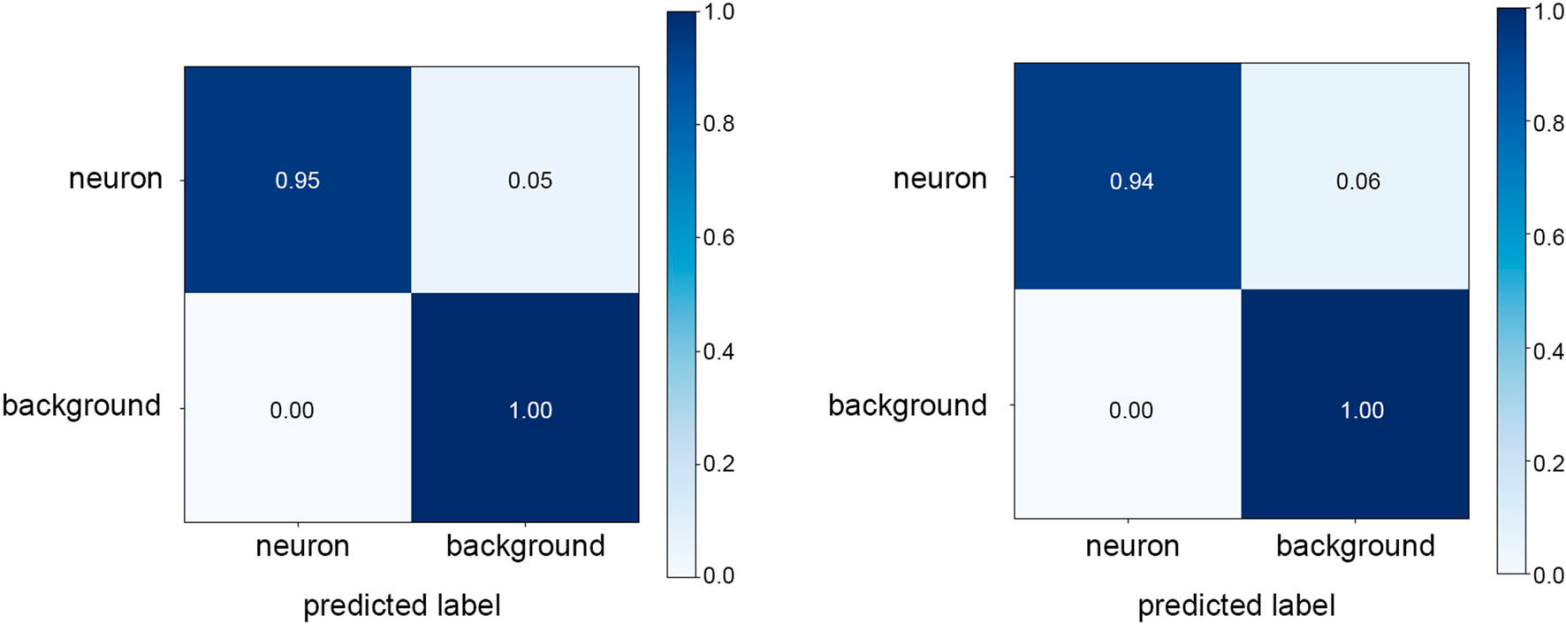
Confusion matrices of instance segmentation model (left) and object detection model (right)

Figure 9 shows the confusion matrix for the instance segmentation and object detection models, respectively. Both models present 100% of true negatives. IS outperforms slightly OD, displaying 95% of true positives compared to 94%. We can conclude that both models never mistake the background with a neuron and are capable in most instances of differentiating the neurons from the background.

## Conclusions and future work

After exhaustive experimentation, and considering the parametric equality and dataset conditions under which the three models were trained, the results leave no doubt that the strategy of using instance segmentation for the detection and characterization of neurons in primary cultures outperforms the strategy of using a combination of object detection and semantic segmentation, both in terms of segmentation and neuronal identification. Tables 4 and 5 highlight the main results obtained in the comparative analysis.

In the case of pixel-level segmentation, we observe that working with instance-level information greatly improves segmentation at pixel-level. This occurs because the information associated with the instance of neurons provides essential characteristics for the model to differentiate pixels belonging to neurons from other similar ones, such as glial cells.

When discussing instance-level classification, it can be observed that both techniques perform at a comparable level. Nevertheless, the metrics indicate a superior performance, once again, of instance segmentation. Figure 8 distinctly illustrates this slight but consistent improvement across the entire curve.

We can conclude that, for both tasks, instance segmentation performs better in both aspects. Additionally, a single instance segmentation model provides us with both segmentation-level metrics, which are very useful in characterizing neurons, and instance-level detection metrics, which are very useful in neuron counting.

**Table 4:** Neuron segmentation comparison: semantic segmentation vs instance segmentation

Semantic segmentation	Instance segmentation
Slightly higher accuracy	Greater number of positive predictions
	Most representative metrics (F1 and IoU) are significantly higher
	The rest of the metrics are also higher

We consider this research a turning point for our subsequent studies, which will now focus on increasing the efficiency of this model for the development of new comprehensive techniques for neuron characterization.

These results can highlight the potential viability of deep learning techniques as a quick and non-invasive way of automatizing neuron counting for medical and research purposes.

As future work, we are already applying the conclusions of this research in the development of a system for the characterization of neurons in primary cultures. Thanks to the conclusions obtained in this work, we have unequivocally selected a technology based on instance segmentation to develop our system. This system will rely on GAN generative networks and extensive specific preprocessing to achieve the best possible results.

**Table 5:** Neuron detection comparison: object detection vs instance segmentation

Object detection	Instance segmentation
Good accuracy	Greater accuracy, recall, and mAP
100% true negatives	100% true negatives
94% true positives	95% true positives
	Most representative metrics (F1 and mAP) are significantly higher

## Limitations

This research, and therefore its results and conclusions, are limited to the complex cultures described in the introduction. These cultures are highly variable over time and include elements such as astrocytes, neurites, and fragments of dead cells. The unique characteristics of these cultures mean that the conclusions of this study cannot be generalized to other cell cultures. Furthermore, this study is confined to purely evaluating two basic strategies, without incorporating any independent improvements or techniques that might be more beneficial for one approach or another.
